# Regulation of the mitochondrial permeability transition pore and its effects on aging

**DOI:** 10.15698/mic2020.09.728

**Published:** 2020-06-22

**Authors:** Damiano Pellegrino-Coppola

**Affiliations:** 1 AI Lab One, Wilhelmina van Pruisenweg 35, 2595 AN The Hague, The Netherlands.

**Keywords:** aging, mitochondria, yeast, mPTP, cell death, adenine nucleotide translocator

## Abstract

Aging is an evolutionarily conserved process and is tightly connected to mitochondria. To uncover the aging molecular mechanisms related to mitochondria, different organisms have been extensively used as model systems. Among these, the budding yeast *Saccharomyces cerevisiae* has been reported multiple times as a model of choice when studying cellular aging. In particular, yeast provides a quick and trustworthy system to identify shared aging genes and pathway patterns. In this viewpoint on aging and mitochondria, I will focus on the mitochondrial permeability transition pore (mPTP), which has been reported and proposed as a main player in cellular aging. I will make several parallelisms with yeast to highlight how this unicellular organism can be used as a guidance system to understand conserved cellular and molecular events in multicellular organisms such as humans. Overall, a thread connecting the preservation of mitochondrial functionality with the activity of the mPTP emerges in the regulation of cell survival and cell death, which in turn could potentially affect aging and aging-related diseases.

## INTRODUCTION

Aging can be described as an alteration of the organism in time, characterized by genetical and biochemical dysfunctional changes that ultimately lead to a physical and mental decline and an increased morbidity. The aging process has been clearly linked to many chronological diseases, representing a great risk factor for the development of cardiovascular diseases, neurodegenerations and cancer [1]. Considering that the human population and the mean life expectancy are growing, there is an increasing urge to identify the molecular mechanisms underlying such process, in order to understand the overlap with diseases [2]. However, today the aging core mechanisms still remain poorly understood, and several theories have been proposed to explain the onset and the progression of aging [3]. Indeed, aging is acknowledged as a complex phenomenon encompassing different molecular and cellular players, described as hallmarks of aging [4]. Moreover, these hallmarks can be connected among them, and for this reason aging can be viewed as a cross-pathway process, highlighting that there could be many concurring causes contributing to aging depending on the individual's background and environmental exposures, and that multiple theories of aging could be right at once, each describing effects of this multifaceted phenomenon from a different perspective. Since genes and pathways associated with these hallmarks have been found as shared with other organisms, aging is also acknowledged as an evolutionary conserved process [5]. Due to the complexity of the aging processes and the average necessary length of the experiments, the budding yeast *Saccharomyces cerevisiae* has been extensively used as model to understand aging at a cellular level [6]. One of the advantages of using yeast is that aging can be dissected in two features, namely chronological and replicative, that can be used as a model for the aging of post-mitotic and proliferative cells, respectively. In particular, chronological aging is related to quiescent yeast cells in stationary phase and their capacity to remain functional over time (chronological lifespan, CLS), while replicative aging is related to the replicative potential of cells as the number of daughter cells a mother cell can generate (replicative lifespan, RLS; reviewed in [6]). Yeast, which has already proven to be an insightful cellular model in many previous studies, such as for cell cycle and for autophagy [7], also proved its value in aging studies, regarding the anti-aging effects of calorie or diet restriction [8], the inhibition of the Tor pathway [9, 10], and by contributing to the discovery of the anti-aging effect of drugs such as spermidine and rapamycin [9, 11]. For these reasons, yeast has been proposed as a screening platform to discover anti-aging compounds [12]. Further, yeast has also been extensively employed as a model to study the relation between oxidative stress and aging [13]. Noteworthy, a main anti-aging thread composed of protective mechanisms that deal with reactive oxygen species (ROS) and oxidative stress can be identified. From this perspective, aging can be connected to the mitochondrial free radical theory of aging (MFRTA; [14]) and how ROS, known mainly as byproducts of respiration, can promote mitochondrial dysfunctions and in turn foster and negatively influence the process of aging. Moreover, it is possible that mitochondria indeed play a key role in aging not just through oxidative stress production and protection, but also with other mechanisms and complexes. One complex that has been increasingly acquiring popularity is the mitochondrial permeability transition pore (mPTP), and it has been extensively connected to aging and aging-related diseases (reviewed in [15, 16]).

In this viewpoint, I will focus on the role of mitochondrial dysfunctions and the mPTP in the context of aging and aging-related diseases, highlighting how evolutionarily conserved molecular mechanisms found in yeast may be used as a guide to understand the molecular mechanisms in humans, and reasoning on the evidence that supports and suggests the mPTP as one of the key regulators of life and death in human cells.

## MITOCHONDRIAL DYSFUNCTIONS AND AGING

The MFRTA is a theory derived from the free radical theory of aging [14, 17]. In this latter, earlier theory, free radicals are pointed out as the executors of oxidative damage addressed to DNA, proteins and lipids, which in turn causes aging and aging-related diseases [17]. Successively, the theory was extended to mitochondria [14, 18], well-known eukaryotic organelles involved in a plethora of cellular events, ranging from metabolism to ATP production, to cell death and more (reviewed in [19, 20]). In particular, it is relevant to highlight that mitochondria are responsible for the oxygen-depended ATP production, well-known as oxidative phosphorylation. During this biochemical process, electron leakage occurs, contributing to the creation of ROS through several mitochondrial sites [21]. Although ROS can act as signaling molecules in the modulation of mitochondrial metabolism and fission/fusion mechanisms (reviewed in [22]), an uncontrolled production is acknowledged to be dangerous, and indeed oxidative stress is a feature associated with many diseases, including cardiovascular diseases, cancer and neurodegenerations [23, 24]. Initially, mitochondria were thought to be highly vulnerable to ROS, with the mitochondrial DNA (mtDNA) as one main target. Mutations in the mtDNA are also a feature of aging [25], and accumulate in different tissues belonging to the heart [26, 27], the skeletal muscles [27], the brain [28, 29] and induce senescence [30]. However, mtDNA mutations are not proven to be drivers of aging or aging-related diseases, as it has been shown that DNA polymerase γ-associated proofreading, mtDNA repair systems and mitophagy are efficient systems that prevent an accumulation of damage to the extent required for aging or pathologies [31, 32] and mutations in the mtDNA need to accumulate to a certain threshold in order to manifest as a disease [33]. This increase in mutation frequency may be caused by enhanced oxidative stress, but experiments on the antioxidant systems have given controversial results (reviewed in [32]), with the paradox of the naked mole rat which has extensive oxidative stress, but also a remarkably long lifespan [34]. However, homozygous *polg*^*mut/mut*^ mice, in which the mitochondrial-specific DNA polymerase γ is mutated and the proofreading activity inactivated, have an accelerated aging phenotype [35]. Further, a DNA damage response-dependent activation of the mTORC1-PGC-1β axis regulates the mitochondrial content contributing to a senescent phenotype [36]. Indeed, the retention of stemness properties has been shown to be the result of an asymmetric division of young mitochondria [37]. Lastly, mitochondrial respiration defects during aging, found to be controlled by epigenetic regulations and not by mtDNA mutations, could be reversed [38]. These results still strongly support a key role of mitochondria in aging, but highlight a different perspective: an increase in ROS and mtDNA mutations, not excluded to be a cause in specific cases, can be generally thought as mechanistically correlated to aging. Overall, this points out that: 1) mitochondria appear as very resilient organelles and 2) the absence of functional mitochondrial activity strongly affects the aging rate.

In yeast, an increase in ROS and oxidative stress has also been correlated to aging and lifespan [39], and there is an increase in mtDNA mutations over time [40]. Noteworthy, yeast during proliferation undergoes an asymmetric division that differentiates the daughter cell from the mother cell, with the former inheriting undamaged cellular components, while the latter retains compromised mitochondria and oxidatively damaged proteins [41, 42]. Further, special yeast mutants called *petite* or rho^0^ lack a functional mitochondrial genome and respiration, and as a result they have a decreased CLS and RLS [43, 44]. However, rho^0^ can undergo metabolic reprogramming, subtelomeric silencing and a mitochondrial adaptation which in turn appears to ameliorate their state and increases their RLS, suggesting that mammalian rho^0^ can also find a way to potentially adapt and pointing out which mechanisms could be studied [30, 45–48]. Indeed, yeast also highlights that the relation between oxidative stress and cellular lifespan may not be so straightforward as initially thought. In fact, the overexpression of the genes coding for the copper-zinc superoxide dismutase and the manganese superoxide dismutase (*SOD1* and *SOD2*, respectively) increases CLS [49]. On the other hand, inactivation of *SOD1* or *SOD2* was found to reduce CLS [50, 51]. Further, inactivation of *SOD1* also dramatically shortens RLS, while inactivation of *SOD2* has no remarkable effect [52]. Regarding catalases, the overexpression of *CTT1* decreases CLS [49], while its deletion has been shown to increase CLS [53]. The notion that the relation between ROS and aging is not so direct is further suggested by hormesis. Yeast cells exposed to sublethal ROS levels during growth have an extended CLS [54]. This supports the role of ROS as signaling molecules that can act as beneficial, a concept being also explored in mammals, including humans, with ROS involved in adaptive, physiological responses to control metabolism and aging [55, 56]. Therefore, the relation between ROS and aging has still to be fully comprehended, in light of the fact that ROS may act as a double-edged sword, therefore it may be that it's not about ROS *per se* but it's related to the level and location of ROS to which cells are exposed [57–59].

## MITOCHONDRIAL PERMEABILITY TRANSITION PORE AND AGING

A plethora of evidence points to the fact that the proper functioning of mitochondria affects the regulation of lifespan. However, neither mtDNA nor ROS have been uncovered as the ultimate causes of aging. Keeping this in mind, it seems plausible to think about additional players, such as the mPTP, that can potentially fit both health and disease-related mechanisms. The mPTP is a non-selective channel situated in the mitochondrial membrane and which, as the name suggests, is responsible for an increase in the permeability of mitochondria allowing the passage of molecules with a mass up to 1500 Da [60]. Due to its complex multimeric nature, the exact composition and structure of the mPTP is currently under investigation (reviewed in [61–64]). It is worth mentioning several components: cyclophilin D (CYPD), regarded as a main controller of the opening of the pore; adenine nucleotide translocator (ANT), which is involved in ADP/ATP exchange and in the functioning of the pore; voltage-dependent anion channel (VDAC), located in the outer membrane; ATP synthase, whose role is being intensively discussed [65–67]. Over the years, the mPTP opening has been linked to different events, such as Ca^2+^ overload and oxidative stress [61–64]. The opening of the pore can hinder mitochondrial metabolism, respiration, and can cause detrimental alterations of the mitochondrial membrane, followed by the release of numerous factors into the cytosol among which Ca^2+^, NAD^+^, ROS, AIF, endonuclease G and cytochrome c, causing a fatal variation in cellular homeostasis which has been linked also to aging (reviewed in [15, 16, 68]). Indeed Ca^2+^, oxidative stress, decrease in ATP levels and membrane depolarization have all been linked to cellular senescence [69–73]. Loss of NAD^+^ is also regarded as a feature of aging and further, the disappearance of NAD^+^ from the cell, subsequent from its release from the mitochondria, can be imputed for instance to the activity of PARP1, involved in the response to DNA damage, or CD38, which has been linked to aging and immune response [15, 74–77]. Lastly, PARP1 hyperactivation, AIF and cytochrome c, are all well known for their role in cell death [78–80].

A mitochondrial permeability transition resembling peculiar aspects of the mPTP has been observed also in yeast, confirming that the molecular mechanisms of aging can be evolutionarily conserved. In yeast, mitochondria can accumulate Ca^2+^, increase ROS generation, release cytochrome c and although the identification of the complete structure of the channel responsible for this permeability is still ongoing, multiple yeast orthologs of the mammalian mPTP have been identified [81–88]. This offers the opportunity to understand the functioning of the pore through another cellular system already acknowledged as a model. In particular, the yeast Nuc1 is a mitochondrial mediator involved in apoptosis, ortholog of the mammalian endonuclease G, and its overexpression promotes cells death in presence of H_2_O_2_ [89]. However, the deletion of the *AAC1/2/3* paralog genes for the adenine nucleotide translocators is able to counteract the Nuc1-mediated cell death. A decrease in survival can be observed, but it is possible that in light of their double role as members of the pore and adenine nucleotide translocators, the deletion of the *AAC* genes impacts cells energetics. Moreover, the isoform of the human adenine nucleotide translocator ANT1 has been shown to confer sensitivity to the pore in presence of H_2_O_2_ [90]. In fact, ANT1 has been shown to increase the sensitivity of the mPTP, but not to be essential, highlighting that other components may play an important role in the opening of the pore [91]. However, the *ANT1* gene is expressed in postmitotic tissues and can be found in the heart and the skeletal muscles, but to a lesser extent also in other organs [92]. Levels are confirmed in the Human Protein Atlas, as *ANT1* can be also expressed in parts of the brain and of other organs [93]. Moreover, *ANT1* has been linked to different diseases, such as progressive external ophthalmoplegia and mitochondrial DNA depletion syndromes (on Online Mendelian Inheritance in Man: 103220; [94]). Over the years, a link between *ANT1*, cardiomyopathy and neurodegenerations has been highlighted [15, 63, 95–98]. Ultimately, a connection between the mPTP, and in particular ANT1, has been established with cell death and in relation to BAX and BCL2 [99–104]. A historical overview on the studies regarding ANT proteins, as well as a collection of structural and functional details over the years has been extensively reviewed [104–106].

Several parallelisms between the human *ANT1* and the yeast ortholog *AAC2* can be made: mutations in *AAC2*, equivalent to the human autosomal dominant progressive external ophthalmoplegia-associated *ANT1* mutations, also led yeast to mitochondrial dysfunctions and affected mtDNA [107]. Further, overexpression of the sole *ANT1* in yeast did not induce cell death [108], as yeast doesn't possess ortholog members of the BCL2 family, but yeast mitochondria are still sensible to BAX-mediated apoptosis [109, 110], and ANT-deficient yeast cells were shown to be resistant to BAX- [99] and HIV-1 viral protein R-induced apoptosis [111]. Indeed, the yeast Aac proteins have been shown to be required for mitochondria permeabilization and cytochrome c release [112, 113].

This preliminary evidence strongly suggests that the ANT1-driven cell death runs not only through the mPTP activity, but can be influenced also by pro-apoptotic factors. Always keeping in mind the differences that exist between a model of a system and the system itself, an evolutionarily conserved pattern between yeast and human can be identified, involving adenine nucleotide translocators, mPTP and cell death. Altogether, this highlights a mechanism that appears to be maintained in the history of eukaryotes, indicating that it could represent a main route in the regulation of cell survival and cell death.

## MITOCHONDRIAL PERMEABILITY TRANSITION PORE AND ANT1 IN HEALTH AND DISEASE MECHANISMS

Recently, an intriguing connection has been made between the mPTP and autophagy: in particular, in both nematodes and mammals a negative influence of mitochondrial permeability on autophagy-induced lifespan expansion was shown [114]. Indeed mitophagy, the specific degradation of mitochondria, is related to mitochondrial membrane potential, with dysfunctional mitochondria - e.g. depolarized as a consequence of damage - being addressed to mitophagy [115]. Mitophagy of mitochondria has also been recently connected to ANT1, independently from its translocase activity [116]. It is possible that in an autophagy-prone mutant, such as the worm *sgk-1*, an over-active mPTP causes damages through components involved in mitophagy, and this could explain why the deletion of mitophagy or fragmentation-related genes, such as the fission gene *drp-1*, had a positive effect on the restoration of lifespan towards normal trends [114].

A lowering of the mitochondrial membrane potential is also associated with calorie restriction (CR; reviewed in [117]), and positive effects of CR have been associated with the inhibition of the mPTP (reviewed in [15]). Further, CR is also known to induce autophagy [118] and has been connected to the promotion of mitochondrial fusion [119–121], proposed as a countermeasure against excessive mitochondrial degradation during autophagy itself [122]. However, it was also found that mitochondrial fission proteins are increased during CR, and an explanation could be related to the role of fission in mitochondrial biogenesis and CR [123]. Depolarized mitochondria were also found in senescent cells [124–126], where autophagy is known to take place [127, 128]. In fact, autophagy was found to be activated in senescence although the details of the interplay between autophagy, senescence and apoptosis have to be clarified [128, 129]. From this scenario, it is possible to start separating a functional mitochondrial depolarization from hyperpolarization as parts of distinct mechanisms. The first associated with CR, autophagy and senescence, while the latter is associated with an increase in ROS production and apoptosis [130, 131], when cells are in a non-cancerous state [132–135]. In agreement, the role of mitochondrial hyperpolarization in apoptosis was also shown in yeast expressing BAX [136]. Further, the link between hyperpolarization, ROS and cell death in yeast has been proven in many different contexts, such as exposure to H_2_O_2_ and cadmium [137, 138], or including thermal stress, as reported and summarized in [139], underlying once again the shared biological patterns between this model system and humans.

**Figure 1:**
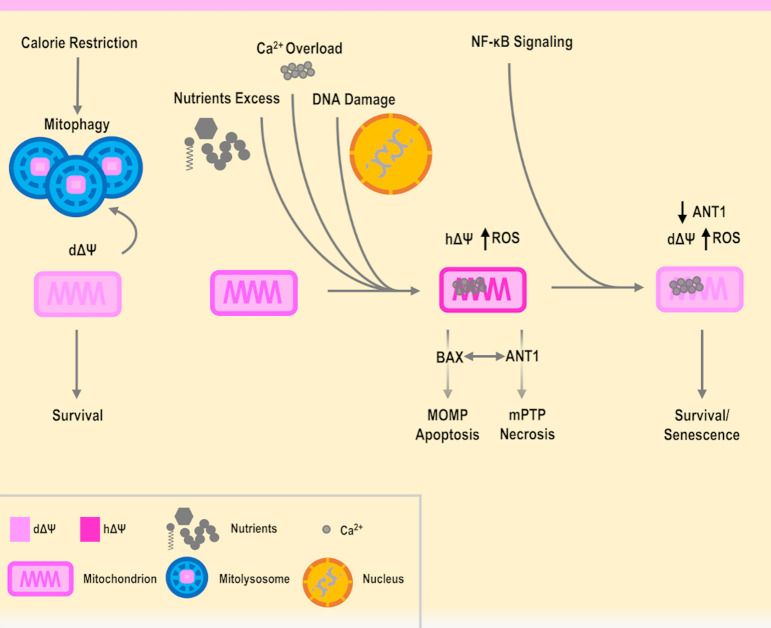
FIGURE 1: The mPTP, a potential hub in the regulation of cell survival and cell death. Calorie restriction features mitochondrial depolarization and mitophagy, promoting survival. During mitophagy, mitochondria are degraded inside mitolysosomes. In contrast, in presence of death stimuli such as excessive nutrients, calcium overload or DNA damage, cells may undergo cell death. The balance between apoptosis and necrosis could be regulated by the mitochondrial membrane potential coupled with the levels of ANT1 activity and the interactions with other players such as BAX. Further, pathways increasing the expression of *ANT1* could foster cell death through the mPTP, while pathways decreasing it (e.g. NF-κB signaling pathway) could favor mPTP closing and cell survival. See the text for details. Δψ, mitochondrial membrane potential with d, depolarized and h, hyperpolarized; MOMP, mitochondrial outer membrane permeabilization; mPTP, mitochondrial permeability transition pore. Organelles, damaged DNA, Ca^2+^ and nutrients (e.g. carbohydrates such as glucose, proteins, fatty acids) are simplified graphical representations.

In addition, it has been shown that senescent cells have an increased level of ANT1 [140], and as seen previously, it is also known that an overexpression of *ANT1* fosters cell death. However, it is also true that senescent cells are resistant to apoptosis and involved in wound healing [140, 141]. Remarkably, the *ANT* genes have a unique interplay with cell death, as the non-pro-apoptotic gene *ANT2* is found in highly proliferative cells such as cancer cells [142, 143], while the overexpression of *ANT1* induces apoptosis in cancer cells [144]. Of note, TGF-β was shown to repress *ANT2* and this contributes to senescence [145–147]. On the other hand, in mice the deletion of *ANT1* has been shown to bring to hypertrophic cardiomyopathy, while inactivating *ANT2* is lethal [148–150]. Indeed, fine-tuning of *ANT* genes and mPTP activity can be both integrated as parts of an interconnected regulatory network involving major cellular pathways in aging, inflammation and diseases, including NF-κB and p53 [142, 151, 152]. In relation to this, NF-κB was shown to downregulate *ANT1* and the activity of mPTP [152, 153]. In a hypothetic scenario, NF-κB could therefore promote senescent cell survival, by lowering mitochondrial potential in a functional and ANT1-dependent manner, allowing to send eventual inflammatory and repairing signals, as platelets where found to be activated by senescent cells [154, 155]. However, this situation revolves around a delicate equilibrium, as excessive stress and damage could cause an increase in the levels of inflammation, further downregulating *ANT1*. For instance, this could avoid the leak of ROS from the mPTP, decreasing the level of ROS needed to play a role in the inhibition of proliferation [59, 156–158], generating an imbalance and fostering a situation where senescent cells drive the origin of cancer cells [159–161]. On the other hand, a forced upregulation of *ANT1* could cause the opposite situation, where cells are prone to cell death, such as in neurodegenerations where the mPTP has been shown to be involved. In this case, NF-κB could promote the survival of neurons, but glial cells foster inflammation and damage [162–164]. Indeed, specific genetic variants could be involved in the modulation of the anti- or pro-death mechanisms. Therefore, ANT1 is a functional and regulatory piece of the mPTP, that is operating as a tap and may influence survival or the execution of a specific type of cell death (**Figure 1**).

## CONCLUSIONS AND FUTURE PERSPECTIVES

The mPTP is regarded as a multimeric complex, of which the complete and definitive structure is currently under investigation. The mPTP has been studied in detail for many years and connected to aging and aging-related diseases, such as neurodegenerations, cardiovascular diseases and cancer. ANT1 appears as a promising candidate to explain the activity of the mPTP, however, it cannot be regarded as exclusive as many other components could have a key role, such as VDAC1 [104, 165, 166]. An additional player involved in the regulation of mPTP is hexokinase II [167–170], which has been placed in the context of functional mitochondrial depolarization, described as a key feature of long-lived animals [171]. Both VDAC1 and hexokinase II have orthologs in yeast (Por1 and Hxk2, respectively), and both have been shown to be involved in cell death [112, 113, 137, 172], in agreement with the fact that the budding yeast *S. cerevisiae* can provide useful information and guidance in the interpretation of the human aging cellular mechanisms. Aging is an evolutionarily conserved process, and shared cellular patterns and pathways are found in eukaryotes ranging from unicellular to multicellular organisms. Evidence accumulated so far can support a beneficial role of functional mitochondria depolarization, as opposed to detrimental hyperpolarization, ROS production and cell death, with the mPTP placed as a regulator in the balance between apoptosis and necrosis (**Figure 1**). Therefore, based on these premises and employing cellular model systems, on one hand it is necessary to proceed with the identification of the parts that compose the structure of the mPTP and which pathways are involved in its regulation. On the other hand, it would be pivotal to employ different omics technologies and computational methods to trace the expression and the activity of the various mPTP components during aging as well as in health and disease, also in relation to metabolism, epigenetics and the microbiome. For instance, starting from RNA-seq data of whole blood samples from over 3000 individuals of different ages, a recent analysis found *ANT1* to be upregulated with aging and nominally significant [173]. Further detailed studies should be planned to consistently confirm this point. Therefore, it is plausible that a threshold could be associated with the mPTP components and activity levels, necessary for a certain aging-related disease to develop, in concert with other molecular cues that govern lifespan and healthspan at the interface between genetic background and environmental exposures.

## Abbreviations:

ANT: – adenine nucleotide translocator;
CLS: – chronological lifespan;
CR: – calorie restriction;
MFRTA: – mitochondrial free radical theory of aging;
mPTP: – mitochondrial permeability transition pore;
mtDNA: – mitochondrial DNA;
RLS: – replicative lifespan;
ROS: – reactive oxygen species;
VDAC: – voltage-dependent anion channel.
